# Three dimensional modelling of the effect of arterial pulse wave velocity and body size on left ventricular geometry

**DOI:** 10.1186/1532-429X-17-S1-O44

**Published:** 2015-02-03

**Authors:** Wareed Alenaini, Declan P O'Regan, Antonio de Marvao, Timothy J Dawes, Wenzhe Shi, Stuart A Cook

**Affiliations:** 1 Natural Sciences, Imperial College London, London, UK; 2 Faculty of Medicine, Imperial College London, Institute of Clinical Science, London, UK; 3 Faculty of Medicine, Medical Research Council Clinical Sciences Centre, London, UK; 4 Department of Computing, Imperial College London, London, UK; 5 Department of Cardiology, National Heart Centre Singapore, Singapore, Singapore

## Background

Left ventricular hypertrophy (LVH) is known to be associated with all-cause mortality and morbidity. There is a strong relationship between LV mass and afterload but the influence of aortic vasculopathy on ventricular geometry is less well understood. Aortic stiffness (AS) is one of the earliest detectable signs of the changes that alter the structure and function of the vessel wall, and may precede the onset of systemic hypertension and ventricular hypertrophy. The aim of this study was to determine the changes in LV geometry associated with aortic pulse wave velocity (PWV) using 3D cardiac imaging of the heart

## Methods

Phase contrast imaging was performed in 403 healthy volunteers using a single axial section to image both the aortic root and descending thoracic aorta. PWV was calculated using Art-Fun (Inserm, Paris) software to detect the systolic upstroke of the flow profile and measure the path length of the aortic arch. High-spatial resolution breath-hold 3D cine imaging was performed of the LV. Each image was segmented and co-registered using prior knowledge from a cardiac atlas. Wall thickness was calculated at each point as the distance between endocardial and epicardial surfaces. Linear regression was used to model to association between variables

## Results

Univariate analysis showed a strong correlation between PWV vs age (r=0.68; P<0.05) and PWV vs mean arterial blood pressure (r=0.414; P<0.05). In multivariate analysis, both age (β= 0.08; P<0.0001) and MAP (β=0.03; P<0.0001) were independent predictors of PWV. The 3D models showed that 55% of the variation in left ventricle wall thickness can be accounted for by a linear model that includes PWV, age, gender, race, and BSA as co-variates. The models demonstrated strong regional associations between PWV and wall thickness which may reflect adaptations to normalize wall shear stress in the LV

## Conclusions

This study of healthy volunteers showed that age is the strongest independent predictor of PWV and that the associated morphological adaptations of the LV may be modelled in 3D. This computational approach to phenotyping may provide insights into the physiological adaptations in the LV associated with vascular function

## Funding

Saudi Ministry of Higher Education.

